# Social Variables Affecting Mate Preferences, Copulation and Reproductive Outcome in a Pack of Free-Ranging Dogs

**DOI:** 10.1371/journal.pone.0098594

**Published:** 2014-06-06

**Authors:** Simona Cafazzo, Roberto Bonanni, Paola Valsecchi, Eugenia Natoli

**Affiliations:** 1 Dipartimento di Neuroscienze, Università di Parma, Parma, Italy; 2 Wolf Science Center, Ernstbrunn, Austria; 3 Azienda USL Roma D, Area Dipartimentale Sanità Pubblica Veterinaria, Rome, Italy; CNRS, France

## Abstract

Mating and reproductive outcome is often determined by the simultaneous operation of different mechanisms like intra-sexual competition, mating preferences and sexual coercion. The present study investigated how social variables affected mating outcome in a pack of free-ranging dogs, a species supposed to have lost most features of the social system of wolves during domestication. We found that, although the pack comprised multiple breeding individuals, both male copulation success and female reproductive success were positively influenced by a linear combination of dominance rank, age and leadership. Our results also suggest that mate preferences affect mating outcome by reinforcing the success of most dominant individuals. In particular, during their oestrous period bitches clearly searched for the proximity of high-ranking males who displayed affiliative behaviour towards them, while they were more likely to reject the males who intimidated them. At the same time, male courting effort and male-male competition for receptive females appeared to be stronger in the presence of higher-ranking females, suggesting a male preference for dominant females. To our knowledge, these results provide the first clear evidence of social regulation of reproductive activities in domestic dogs, and suggest that some common organizing mechanisms may contribute to shape the social organization of both dogs and wolves.

## Introduction

In animals living in mixed-sex social groups the mating and reproductive outcome is often determined by the simultaneous operation of different mechanisms such as intra-sexual competition, mating preferences as well as sexual coercion. Male intra-sexual competition has been the primary focus of researchers investigating the factors influencing mating and reproductive success in vertebrates [1], [2]. In general, they have shown that in several species dominant males have a higher mating success than subordinates (e.g. [3], [4], [5], [6], [7]), though the relationship between male dominance rank and mating success is very complex and can vary between different species, social structures and mating systems [8], [2], [9], [10], [11], [12], [13]. Moreover, males can adopt various strategies to try to increase their mating success. For example, at one extreme, some males, can coerce reluctant females to mate with them [14], [15], [16,]. Sexual coercion can occur: 1) when a male uses his superior speed or strength to catch and physically restrain a female while he copulates with her by force (forced copulation); 2) when a male repeatedly attempts to copulate with a female inducing her to mate, since trying to refuse him entails costs to her (harassment); 3) when a male punishes a female that refuses to mate with him (intimidation) [16]. Moreover, males can also coerce females indirectly, i.e. by attempting to decrease the relative mating success of other males through mate guarding, punishment of females that attempt to mate with other males, copulation interference and even infanticide [17], [18]. It has been observed that the females' willingness to mate may be inhibited by coercing attempts displayed by males during courting [19]. However, at the opposite extreme, there is also evidence that in some species of mammals males are able to increase their chances of mating with a particular female by developing an affiliative relationship with her [20], [21], [22], [23].

Female mate choice may actually be the other major factor interacting with male-male competition to generate mating outcomes. Since the investment of females in reproduction is usually higher than that of males, and their reproductive success depends on male quality (whereas male reproductive success depends on the number of fertilized females), they are expected to be more selective than males in mate choice [24], [25], [26], [27], [28]. The interaction between female mate choice and male-male competition may affect mating outcome either by reinforcing the success of the most dominant males (e.g.[6], [29], [30]), or by acting in opposition to dominant males [31], [32], [33], [34], [35], [36], [37].

The possibility of males demonstrating mate preferences based on specific female characteristics has received less attention. Although females tend to be choosier than males in selecting mates, sexual selection theory predicts that males should also be choosy 1) when females differ in quality, 2) when males seek long-term partners, and/or 3) when they allocate resources to females or to their offspring [38]. Since competition among males for mates can be costly, they should prefer to mate with females likely to produce the highest number of surviving offspring [8], [39]. Indeed, male preferences have been documented in various taxa for older females with more experience [40], for larger and more fecund females [41], [42], [43], [44], [45], [46], [47], for dominant females ([48], [49], [50], [51]; but see [52]), and for females exhibiting superior parental care [53]. Mutual mate choice might be most common in monogamous species where both sexes have similar parental roles [54], [55], [56], but there is also some evidence for mutual mate choice in promiscuous species [57].

In this study, we investigated the social variables affecting both male and female mate preferences in a pack of free-ranging dogs (*Canis familiaris*), i.e. those domestic dogs whose movements, activities and reproduction are not constrained by human beings and that, according to recent studies, may actually represent the most numerous category of domestic dogs in the world [58]. In areas where they have access to abundant food resources directly or indirectly provided by human beings, free-ranging dogs can live in stable packs formed by multiple breeding individuals of both sexes [59], [60], [61], [62], [63], [64], [65], [66], [67], [68], [69], [70]. There are several claims in the scientific literature that, due to the effects of domestication on their behaviour, free-ranging dogs are unable to form structured social groups and retain very little of the social organization of wolves (*Canis lupus*), that are their closest living relatives [71], [72], [73], [74]. In particular, the presence of multiple breeding individuals in dog groups seems in sharp contrast with the structure of wolf family groups, usually comprising a single dominant breeding pair and a number of subordinate non-breeding helpers [75], [76], [77], and has led researchers to conclude that domestic dogs lack any social regulation of reproductive activities [72], [74]. However, recent studies [65], [68], [70] have demonstrated that free-ranging dogs show a complex social organization characterized by age-graded dominance hierarchies in which males tend to be dominant over females of similar age, although females often are dominant over younger males. Furthermore, dominance relationships are expressed both in agonistic interactions and in affiliative greeting ceremonies [65] and, as in wolves [78], older dominant individuals usually lead the collective movements of the pack [68], [70]. Nevertheless, it remains to be demonstrated whether dominance relationships in dogs result in some kind of social control of reproduction within the group, as has been observed in wolves (see references above).

Unlike wolves, most free-ranging dogs exhibit a promiscuous mating system in which both males and females mate with multiple partners (e.g.[79], [80], [81]). Due to the differential costs of reproduction in promiscuous species [23], [82], [83], [84], females should mate preferentially with high quality males, while males should attempt to mate with as many females as possible. Nevertheless, some evidence suggests that mutual mate choice might affect mating and reproductive outcome in domestic dogs. Several authors reported that bitches mate with certain males while refusing others [79], [80], [81], [85], [86], [87], [88]. At the same time, males are attracted more to females in their second or subsequent oestrous periods than they are to females in their first oestrus [86].

There is also some evidence that free-ranging dogs may adopt different strategies in order to gain mating opportunities. For example, in some cases, male dogs were observed to show aggression towards oestrous females before mating with them, and to use force to gain mating, providing support for the occurrence of male sexual coercion in this species [80], [81], [86], [87]. Both increased aggression between males during courting of receptive females and copulations disrupted by the interference of other males have been observed in free-ranging dogs [65], [79], [80], [87]. Furthermore, affiliative relationships have been observed between dogs belonging to the same pack [67], [77], raising the question whether, as in other mammalian species (see references above), inter-sexual affiliative interactions may also be functional in order to gain mating opportunities.

Here, we aimed first at describing the mating and reproductive pattern observed in the pack studied. Then, we aimed at investigating the social variables affecting mating outcome by answering the following main questions: are female and male mate preferences influenced by the social rank and leader role of mating partners? Are high-ranking dogs and habitual leaders of the pack the most successful in mating and reproduction? Which strategy is more efficient in increasing the mating chances of males: “to be friendly” or “to be aggressive” towards females? Does the reproductive success of females influence male mate preferences?

As in other species of mammals, we can predict that high-ranking male dogs should gain priority of access to oestrous females through direct competition with other males during courtship and/or through female choice. We aimed at assessing the relative importance of both these mechanisms. If affiliative relationships affect female mating preferences, then we expect that males showing a higher frequency of affiliative behavioural patterns towards oestrus females would have a higher copulatory success. Conversely, if male aggression to females is an effective mating strategy, then males showing a higher rate of aggression to oestrus females should have a higher copulatory success.

Differences in mates' attractiveness may be due to differences in their reproductive success that, in some mammal species, has been shown to be positively correlated to social rank [89], [90], [91], [92], [93], [94], [95], [96] and age [90], [97], [98], [99], [100]. Therefore we can predict that higher-ranking, older, and more experienced females should be more attractive to males.

Finally, on the basis of our results, we aim at comparing the mating system of free-ranging domestic dogs to the mating system of wolves.

## Materials and Methods

### Ethics Statement

This study complies with the Italian regulations regarding the ethical treatment of stray domestic dogs.

In April 2006 the Veterinary Public Service of Rome, in collaboration with the Municipality of Rome, started a management project of the dog population with the aim of capturing and sterilizing all the animals in the area. Therefore, during the last month of the study and the following 4 months a number of animals belonging to the pack (4 adult males, 1 juvenile male, and 2 juvenile females) were captured and sterilized. All dogs were captured by using dart guns filled with anaesthetic. After that, animals were immobilized and transported to a veterinary clinic for sterilization. During this time a full check-up was given to the animals including age assessment through dental inspection. After about 4–6 days of permanence in the clinic animals were released in the area. None of the animals was sacrificed for the purposes of our study.

Research permission to conduct the observational study as well as to handle the animals during the immobilization phase was granted by the authorizing body i.e. the Veterinary Public Service of Rome.

### Study area

The study was carried out in a suburban environment at the southwest periphery of Rome, in Italy (41°50′N, 12°23′W; elevation: about 60 m above sea level) covering about 300 ha. The area comprised a northeast sector occupied by part of a nature reserve called “Tenuta dei Massimi”, and an urbanized sector (not densely populated) in the southwest.

The habitat in the reserve consisted mainly of open grasslands with interspersed wooded areas (for a more detailed description see [67]). Dogs had free access to virtually every part of the study area. Nevertheless, all members of the pack studied mainly frequented the reserve where the dense vegetation of the wooded areas offered resting sites and good shelter for the animals, especially for lactating females and their puppies. However, they frequently approached a central road crossing the study area, especially in the early morning, to feed on the food (mainly meat from a slaughterhouse) brought every day by volunteer dog caretakers.

### Subjects

The dog pack studied belonged to a population of about 100 adult animals inhabiting the study area. Although all pack members subsisted mainly on the food provided by humans, they were not socialized to humans, and could move and breed freely. All individuals who had stable social relationships, who were observed interacting friendly also outside the breeding periods and who spent most of the time together in the same area were considered to be pack members. They travelled, fed and defended resources together.

All animals were medium-large sized mongrels, and there was not a recognizable predominant breeding type [70]. They were individually recognized by coat colour and pattern, hair length and body size, and were sexed by genital morphology (for a detailed description, see [65], [67], [68]).

Behavioural observations of the pack began in April 2005 and lasted until the end of May 2006, but all females and their offspring were monitored until the end of June 2007. Dogs were followed on foot and their behaviour was observed with binoculars, when necessary, and noted by hand. We had direct knowledge of age in dogs 2 yr and younger. The ages of the remaining dogs were roughly estimated by assessing body size and general appearance (e.g. white hair on the muzzle) as well as tooth wear (e.g. [101]) and eruption [102] during capturing and immobilization procedure. Owing to deaths, births and disappearance of some individuals during the period of observation, pack size ranged from 25 to 42 individuals, although most statistical analyses were carried out on 27 individuals that were members of the pack throughout the study period, plus 4 dispersed females and 7 non-pack males attracted in the area by oestrous females. All males who were observed interacting with pack members only during the breeding season were considered as non-pack members. Some of these males were observed in the area only during the estrous period while some others belonged to neighboring packs.

### Behavioural observations during oestrous

Out of 14 females that were present at the beginning of the study, ten (6 adults, 1 sub-adult and 3 juveniles) went into oestrous during three seasons (autumn, winter and spring). We observed the oestrous period of these females using both focal subgroup sampling (for a total of 85.25 hours of recording; mean number of hours per female ± SD: 8.53±5.80) and *ad libitum* methods (387.94 hours of observation; [103]). We recorded the behaviour of all courting males (16 males belonging to the pack and 7 non-pack males) that were present within 15 metres from the oestrous female.

An oestrous female was characterized by a swollen vulva and by vaginal bleeding. Observations began when one or more males tentatively approached the female to sniff and try to mount her. The first day in which a female allowed a male to mount and to copulate was noted as the beginning of full oestrus. Observations continued throughout the period in which the female accepted the mount-attempts, and stopped when the female refused to allow males in her company to mount and to copulate for two or more successive days. The first of these days was recorded as the end of oestrus [104].

During each oestrus we recorded the following behaviours (all occurrences sampling methods [103]): 1) mounts (a male attempts to mount a female from the rear but this does not result in the formation of a copulatory tie); 2) copulation ties (a male mounts the female and copulates with formation of the copulatory tie); 3) refusals (a female refuses the attempts to mount by males either through attacking/chasing them, termed “aggressive refusals” henceforth, or through sitting down/moving away, termed “non-aggressive refusals” henceforth); 4) interrupted mounts (a male interrupts the mount between the oestrous female and another male by attacking him and, in this way, he separates the couple); 5) affiliative (e.g. tail wagging, grooming, passive contact) and 6) aggressive (e.g. baring of the canines, snarling, growling, barking) behavioural patterns displayed by males towards females; 7) female approaches (an oestrus female approaches a courting male by moving from a distance greater than 1 meter to a distance smaller than 1 meter from him); 8) female leaves (an oestrus female leaves the proximity of a courting male, by moving from a distance shorter than 1 meter to a distance greater than 1 meter from him; 9) agonistic behavioural patterns (i.e. aggressive, dominance and submissive behavioural patterns; for a description see [65]) displayed by males towards other males within 15 metres from the receptive females.

The rate of both mount attempts and interrupted copulations received by each female was recorded in order to obtain a measure of male mating effort and therefore it was a proxy for male mate preferences.

The approaches displayed by oestrus females towards courting males were regarded as affiliative signals aimed at decreasing the distance from males. In order to use this measure as an indicator of female preferences for particular males, we calculated the “net number of approaches” by subtracting the total number of times the female left the close proximity of a given courting male (female leaves) from the total number of times she approached him.

The individual measures of all behavioural patterns were corrected for time spent within 15 meters of the oestrus female in the case of males, and for total hours of observation during oestrus in the case of females.

### Reproductive success

Female reproductive success was defined here as the number of pups of a given female that survived until sexual maturity (8 months old). Reproductive success was scored as 0 in the following cases: 1) if none of the puppies of a breeding female survived until sexual maturity; 2) if a female in reproductive age (at least 8 months old) was never observed going into oestrous during the study period.

### Dominance rank and leadership

By using data on the direction of submissive behavioural patterns observed both during greeting and agonistic interactions, we could arrange all pack members in a linear dominance hierarchy whose details are described elsewhere [65]. For the aim of present study, individuals were assigned a standardized dominance rank by distributing ranks evenly between the highest rank (standardized rank +1) and the lowest rank (standardized rank -1).

In order to measure the tendency of individuals to lead pack movements in correspondence of pack activity shifts we used the “leadership score” reported for the same pack in [68]. Note that we did not consider collective movements observed during oestrus times in the assessment of leadership. We defined a “leader” as the first dog that started to move in a direction followed by at least two companions within ten minutes (see [68] for more details).

A few months after the beginning of the study, 4 adult pregnant females dispersed from the pack to give birth elsewhere (Table 1). Although we collected data during the oestrous periods of these females, the agonistic dyadic interactions and the collective movements involving them were not sufficiently numerous to assess either their dominance rank or their leadership score. The hierarchical rank and the leadership score of non-pack males in their respective packs were also not known.

**Table 1:** Identity, age class at the end of the study (young: 6 mo to 1 yr; sub-adult: 1 yr to 2 yr; adult: more than 2 yr), gender, pack membership, standardized dominance rank, leadership score, mating pattern and denning location (inside the core area, inside the home range of the pack and at other places) of the dogs studied.

Dog identity	Age class-Gender	Pack membership	Full Oestrous period	Standardized Rank	Leadership score	Mating partners (number of observed copulations)	Male mating/Female reproductive success	Denning site location
MER	Adult male	Resident	-	1,000	1,000	MAY(4),ISO(1),BAG(1),GIN(1)	7	-
GAS	Adult male	Resident	-	0,920	0,667	MOR(2), PIS(1)	3	-
PIP	Adult male	Resident	-	0,840	0,421	MOR(1), CUC(2)	3	-
LEO	Adult male	Resident	-	0,760	0,294	-	0	-
GOL	Adult male	Resident	-	0,680	0,524	PIS(1)	1	-
LAN	Adult male	Resident	-	0,600	0,455	-	0	-
MAY	Adult female	Resident	Early May ‘05	0,520	0,800	MER (4)	7	Core area
NAN	Adult female	Resident	-	0,440	0,545	-	0	Home range
ISO	Adult female	Resident	Early July ‘05	0,360	0,647	MER(1), MSI(1)	2	Core area
DIA	Adult female	Resident	Mid-Jan. ‘06	0,280	0,500	SIM(1)	0	
SIM	Subadult male	Resident	-	0,200	0,250	DIA(1)	1	
PON	Subadult male	Resident	-	0,120	0,286	-	0	
SEM	Subadult male	Resident	-	0,040	0,231	-	0	
KIM	Subadult male	Resident	-	-0,040	0,118	-	0	
MOR	Subadult female	Resident	Late Jan. ‘06	-0,120	0,176	PIP(1), GAS(2)	1	Home range
HAN	Young male	Resident	-	-0,200	0,000	-	0	
CUC	Young female	Resident	Mid-Mar.‘06	-0,280	0,000	PIP(2), PTE(1)	0	Home range
MAM	Young male	Resident	-	-0,360	0,000	-	0	
GON	Young male	Resident	-	-0,440	0,000	-	0	
DOT	Young male	Resident	-	-0,520	0,000	-	0	
GRE	Young female	Resident	-	-0,600	0,000	-	0	
BRO	Young male	Resident	-	-0,680	0,000	-	0	
EOL	Young male	Resident	-	-0,760	0,000	-	0	
EMY	Young female	Resident	-	-0,840	0,000	-	0	
MAG	Young female	Resident	-	-0,920	0,000	-	0	
PIS	Young female	Resident	Mid-Apr. ‘06	-1,000	0,000	GOL(1), GAS(1)	1	Home range
BAG	Adult female	Dispersed	Late July ‘05	-	-	MER(1),SIR(1)	3	Other place
GIN	Adult female	Dispersed	Mid-Sept. ‘05	-	-	MER(1)	2	Other place
RIC	Adult female	Dispersed	Late May ‘05	-	-	-	1	Other place
MOL	Adult female	Dispersed	Mid-Nov. ‘05	-	-	-	0	
BOS	Adult male	Non-pack	-	-	-	-	0	
TRU	Adult male	Non-pack	-	-	-	MOL(1)	1	
MSI	Adult male	Non-pack	-	-	-	ISO(1)	1	
MIS	Adult male	Non-pack	-	-	-	-	0	
ISM	Adult male	Non-pack	-	-	-	-	0	
SIR	Adult male	Non-pack	-	-	-	BAG(1)	1	
PTE	Adult male	Non-pack	-	-	-	CUC(1)	1	

Male mating success  =  number of copulation ties. Female reproductive success  =  number of puppies survived to maturity.

Both standardized dominance rank and leadership score of the dogs studied are reported in Table 1.

### Statistical analysis

In order to explain variation in male copulation tie rate and in the rate of refusals that each male received by females, we used the following predictor variables: 1) male standardized dominance rank; 2) male leadership score; 3) male rate of aggression towards oestrus females; 4) net rate of approaches within 1 meter received by oestrus females; 5) rate of male affiliative behavioural pattern towards oestrus females. Since we expected that several of these variables could be correlated, we applied a principal components analysis (PCA) in order to replace them with new uncorrelated component variables, linear combinations of the original variables, called principal components or factors. Then, we ran two different general linear models using “male copulation tie rate” and “rate of received refusals” as dependent variables respectively, and using the first two factors of the PCA, that explained most variation in the data, as predictor variables. Male mount attempt rate was highly and positively correlated to the rate of received refusals (r = 0.87, n = 16, P = 0.0001) that is a consequence of the fact that most mount attempts performed by males were usually followed by a clear refusal by oestrus females. So, we decided to use in the analysis only the rate of received refusals in order to emphasize the active role of females in choosing partners. We also calculated the “proportion of male mount attempts that were refused by females” and found that this further dependent variable was highly and negatively correlated to “copulation tie rate” (r = −0.83, n = 16, P<0.0001). This shows that copulation ties often occurred when mount attempts were not refused by females, and thus they were strongly affected by female preferences. Conversely, the copulation tie rate was not significantly correlated to the rate of received refusals (r = −0.38, n = 16, P = 0.14).

In order to investigate whether male mate preferences were affected by female dominance rank and experience, we first ran a PCA using, this time, female standardized dominance rank, female leadership score and female age as original variables. Then, we used Pearson's correlation to seek for a relationship between the first factor of the PCA, that explained alone most variation in the data, and the following variables: rate of copulation ties received by each female, rate of aggressive and non-aggressive refusals showed by females towards males, rate of received mount attempts, and proportion of received mount attempts that were interrupted by males. The latter variable was calculated as the number of received mount attempts that were interrupted by competing males divided by the total number of mount attempts that each female received by males. For these correlations we considered only the female observed during oestrous (n = 6) for whom both dominance rank and leadership score were known. We did not apply Bonferroni correction due to our very low statistical power (see [105]).

We also tested whether female reproductive success was related to female dominance rank and experience by running a general linear model with female reproductive success as dependent variable and the first factor of the previous PCA as predictor variable. For this analysis we considered both the females observed going into oestrous (n = 6) and the females that were never observed going into oestrous (n = 4). This was done in order to make a comparison with the reproductive system of wolves, where usually subordinate females do not reproduce [77].

Model residuals were tested for normality using the Kolmogorov-Smirnov test. All statistics were performed using Statistica 7.1 edition (StatSoft Italy s.r.l. 2005).

## Results

### Descriptive statistics

During the period of the study 4 females were never observed going into oestrous although they had reached the age of sexual maturity. The other 10 females went into oestrous between May 2005 and April 2006 (Table 1) and reproductive synchrony among them was low. During their oestrous periods all females were courted by males belonging to the pack (n = 16) as well as by some non-pack males (n = 7). Most females exhibited selectivity by approaching some males and readily permitting them to mate, whereas they avoided or even attacked other males if they attempted to mount them. The number of non-pack males and the time that they spent courting females were, unfortunately, too low to compare their mating success with that of resident males. Nevertheless, some females approached non-pack males and copulated with them. In fact, out of 19 copulatory ties observed throughout the period of the study, 4 were performed by non-pack males. In particular, 4 non-pack males copulated with one high-ranking adult female, one low-ranking young female and two adult dispersed females respectively. These females also copulated with resident males, except one dispersed female. All other females were observed copulating only with resident males. Moreover, it is worth noting that the highest-ranking female was the only one who was observed copulating more than once with only one partner, i.e. the alpha male.

All resident males who were observed copulating more than once had more than one mating partner. In fact, since females went into oestrous at different times, males had the opportunity to court all of them. Overall, the mean number ± SE of mating partners observed was 0.63±0.29 for resident males and 1.40±0.22 for oestrus females.

The pregnant females who dispersed from the pack before giving birth (n = 4) did not interact with other members of the pack during late pregnancy, parturition and lactation (if they had surviving pups). Among the resident pregnant females (N = 6), only high-ranking ones gave birth inside the core area of the pack, whereas low-ranking females delivered outside the core area borders, although inside the home range. The core area was the sector of the pack's home range that was most intensively used by pack members, and where the main feeding sites and resting places were located. The home range was considered as the minimum convex polygon connecting the outermost sightings of pack members.

Dispersed females were never observed coming back to the pack. Two of them (RIC and MOL) joined another pack of 12–15 dogs; another one (GIN) formed a small pack with her pups and two males abandoned in the area; the last one (BAG) remained alone with her pups until the end of the study.

Table 1 reports the mating partners of each individual, their identities, the oestrous periods of each female, as well as the number of copulation ties performed by males and the reproductive success of females.

### Variables affecting male copulation rate and refusals received by females

**Figure 1:**
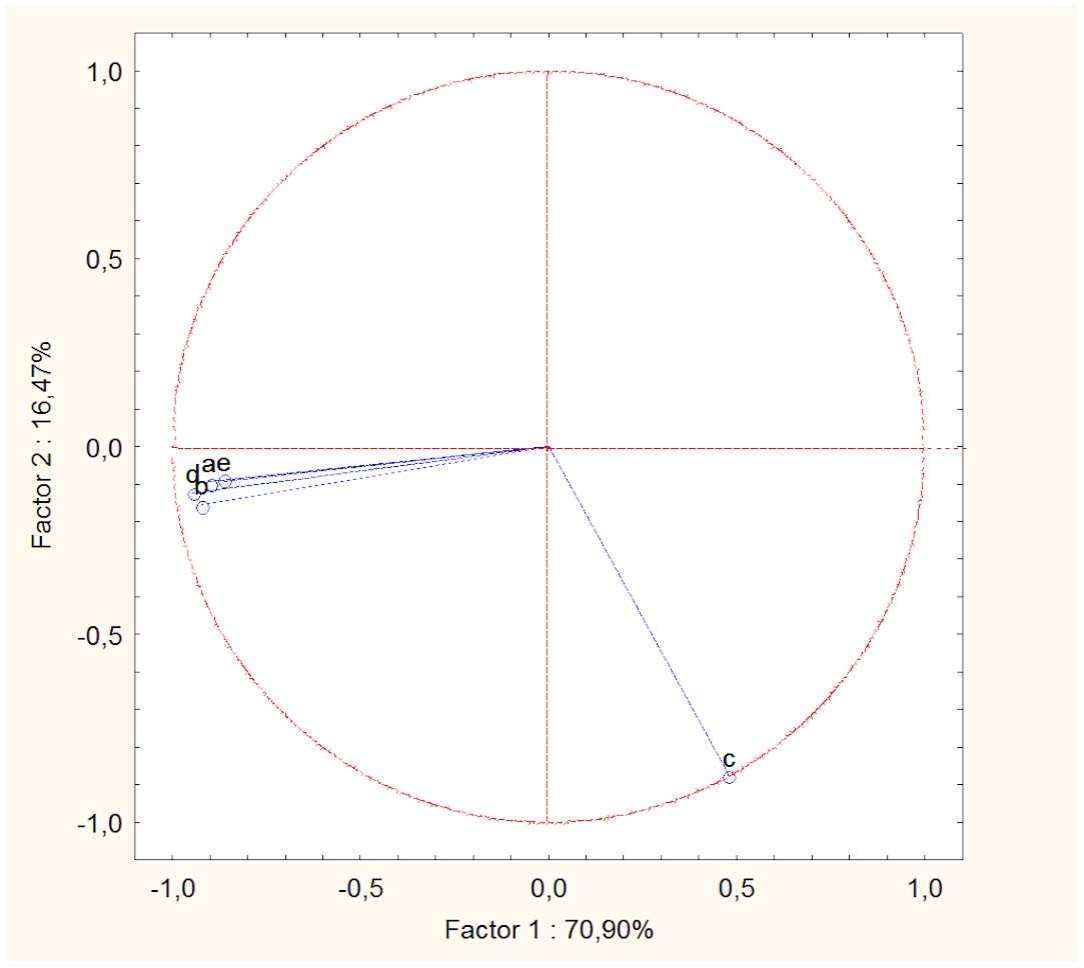
Results of the principal components analysis (PCA) for resident males. The circle shows the correlation between the original variables and the two new components. a  =  male standardized dominance rank, b  =  male leadership score, c  =  rate of aggression displayed by males towards females, d  =  rate of the net number of approaches within 1 metre that males received by females, e  =  rate of affiliative behaviour displayed by males towards females. Pearson correlations between factor 1 of the PCA and the original variables were: −0.90 (a), −0.92 (b), 0.48 (c), −0.95 (d), −0.87 (e); Pearson correlations between factor 2 and the original variables were: −0.09 (a), −0.15 (b), −0.88 (c), −0.12 (d), −0.09 (e).

The first factor of the PCA explained alone 70.90% of the total variance in the data, and was highly and negatively correlated with four of the original explanatory variables (male dominance rank, male leadership score, net rate of approaches that males received by oestrus females, rate of male affiliative behaviour towards oestrus females). Conversely, it was positively correlated with the rate of male aggression towards oestrus females (Fig. 1). This variable was indeed the only one to show a high (and negative) correlation with the second factor of the PCA which explained 16.47% of the total variance. So, males characterized by high negative scores on the first factor were high-ranking individuals who often behaved as leaders, who showed a high rate of affiliative behaviours towards oestrus females and a low rate of aggressive behaviours towards them, and who were frequently approached within 1 metre by females. Conversely, dogs with high negative scores on the second factor displayed a high frequency of aggression towards oestrus females (Fig. 1).

**Figure 2:**
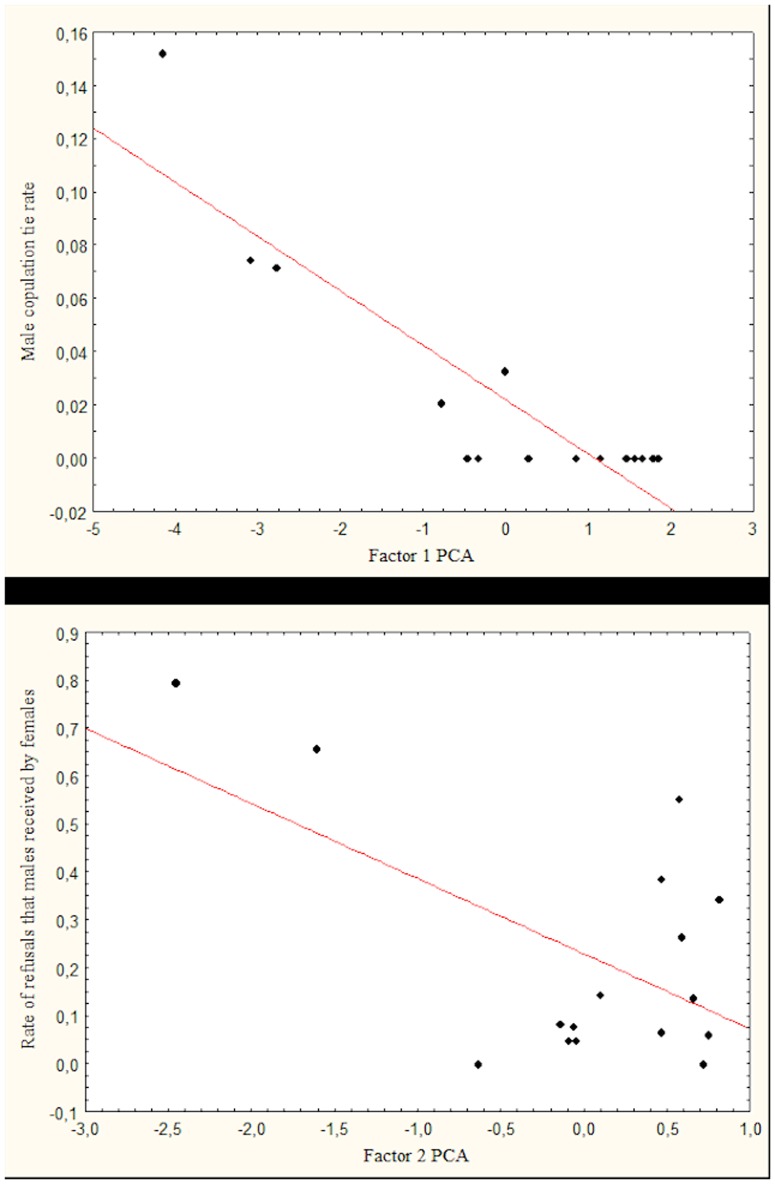
Results of the general linear models developed for males. (a) The relation between ‘male copulation tie rate’ and the first factor of the PCA. High negative values on factor 1 indicate high-ranking males who often led the pack, who were frequently approached within 1 metre by females and who displayed both a high rate of affiliative behaviour and a low rate of aggressive behaviour towards females; (b) The relation between “rate of refusals that males received by females” and the second factor of the PCA. High negative values on factor 2 indicate a high rate of aggressive behaviour displayed by males towards females.

The general linear model developed for ‘copulation tie rate’ was significant (R = 0.91, F_2,13_ = 30.13, P = 0.000013) and showed that the first factor of the PCA was a significant predictor of the copulation success of males (linear coefficient ± SE  =  −0.020±0.003, t = −7.655, P = 0.000004; Fig. 2a): high-ranking males who more frequently interacted affiliatively with oestrus females were more likely to copulate with them. Conversely, the second factor of PCA, was not a significant predictor of the dependent variable (linear coefficient ± SE  = −0.007±0.006, t = −1.289, P = 0.22).

The general linear model developed for ‘refusal rate’ was also significant (R = 0.69, F_2,13_ = 5.87, P = 0.015), and showed that the second factor of the PCA was a significant predictor of the dependent variable (linear coefficient ± SE  = −0.157±0.055, t = −2.845, P<0.014; Fig. 2b): male dogs who were more aggressive towards oestrus females were also more likely to be refused by females. The first component of PCA tended to be positively related to the ‘refusal rate’, although this effect was not significant (linear coefficient ± SE  = 0.051±0.027, t = 1.911, P = 0.078).

### Male mate preferences in relation to female age, dominance rank and leadership

**Figure 3:**
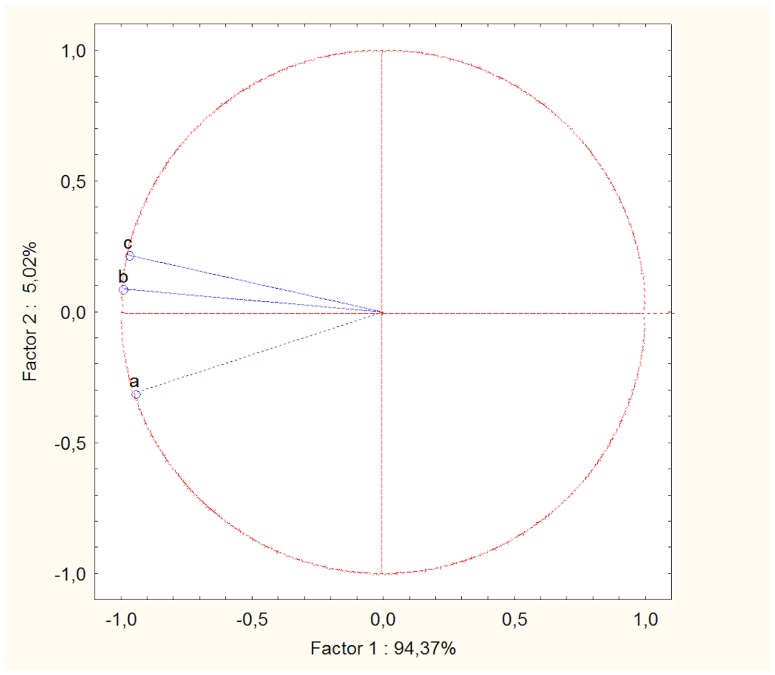
Results of the principal components analysis (PCA) for females. The circle shows the correlation between the original variables and the two new components. a  =  female standardized dominance rank, b  =  female leadership score, c  =  female age. Pearson correlations between factor 1 of the PCA and the original variables were: −0.95 (a), −0.99 (b), −0.97 (c); Pearson correlations between factor 2 and the original variables were: −0.31 (a), 0.09 (b), 0.21 (c).

**Figure 4:**
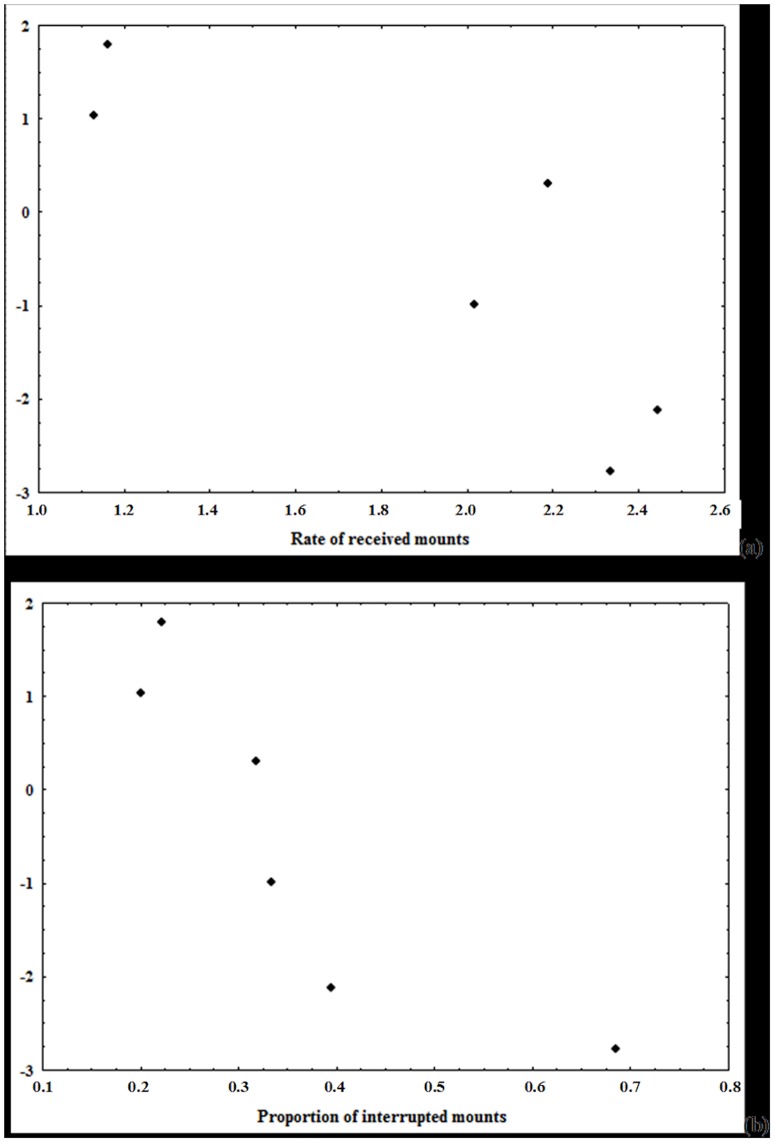
Correlation between the first factor PCA developed for females and: (a) rate of received mounts; (b) proportion of interrupted mounts. See text for additional explanations.

The first factor of the PCA developed for bitches explained alone 94.37% of the total variance in the data and was highly and negatively correlated with all the original explanatory variables (i.e. female dominance rank, leadership score, age; Fig. 3): females with high negative scores on this factor were old, high-ranking individuals who often behaved as leaders. The first factor of PCA was highly and negatively correlated to the received mount attempt rate (r = −0.86, n = 6, P = 0.03; Fig. 4a) and to the proportion of interrupted mounts (r = −0.87, n = 6, P = 0.03; Fig. 4b). High-ranking, old females, who frequently led the pack, were mounted more frequently by males than low-ranking, young females who rarely led the pack. Moreover, males interrupted the mounts between other males and high-ranking, old females more frequently than the mounts between other males and low-ranking, young females. However, we did not find a significant correlation between the rate of received copulation ties and the first component of the PCA (r = 0.37, n = 6, P = 0.46). Finally, the rate of female aggressive refusals was highly and negatively correlated to the first factor of PCA (r = −0.96, n = 6, P = 0.002), while a positive a significant correlation was found between the rate of female non-aggressive refusals and the same factor (r = 0.83, n = 6, P = 0.04). So, aggressive refusals were displayed mainly by old, high-ranking females, whereas juvenile low-ranking females rejected unwanted males mainly in a non-aggressive manner.

### Female reproductive success

**Figure 5:**
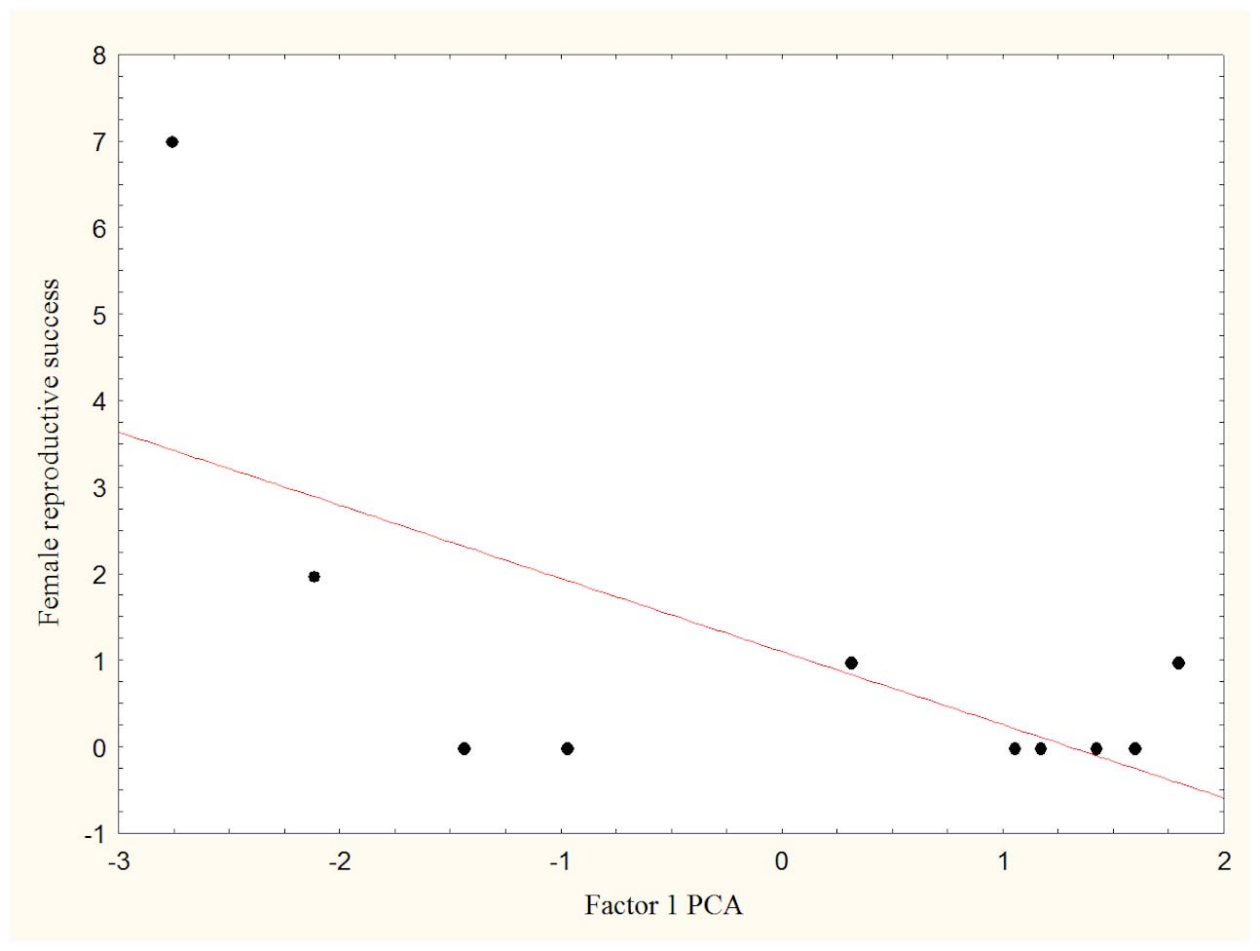
The relation between ‘female reproductive success' and the first factor of the PCA developed for females. Reproductive success has been assessed as number of puppies survived to maturity. High negative values on factor 1 indicate high-ranking and old females who often led the pack.

The general linear model developed for ‘female reproductive success’ was significant (R = 0.65, F_1,8_ = 5.89, P<0.04), and revealed that the first component of the PCA (that was negatively correlated to female age, dominance rank and leadership score) was a significant predictor of female reproductive success (linear coefficient ± SE  = −0.85±0.35, t = −2.43, P<0.04; Fig.5). Therefore, high-ranking, old females who frequently behaved as leaders enjoyed higher offspring survival than low-ranking, young females who rarely or never behaved as leaders.

## Discussion

The present results suggest that in our pack of free-ranging dogs both male copulation success and female reproductive success were strongly influenced by individual social status, with high-ranking dogs (particularly the highest-ranking male and female), that usually led the pack movements, being more successful. Our data also indicate that mating outcome may be due to an interaction between intra-sexual direct competition and mate preferences for high-ranking individuals, with the latter affecting the mating outcome by reinforcing the effect of the former (i.e. the success of high-ranking individuals).

### Intra-sexual competition and mate preferences

In our dog pack, the dominance rank of males achieved through competition outside the oestrous period [65] affected the direct competition among males during courtship: subordinate males had lower copulation success partly because they were intimidated by dominant males who often did not need to attack to keep them away from females. Nevertheless, our results indicate that male intra-sexual competition also affected female preferences. Our data on male-female affiliative interactions during oestrus suggest that bitches prefer to mate with high-ranking males. In particular, oestrus bitches approached high-ranking males at a significantly higher rate than they approached low-ranking males, thus showing a preference for maintaining a close proximity with dominant males. Moreover, high-ranking males were also those who showed more frequently affiliative behaviour towards oestrus females, and this strategy apparently was successful because they achieved a higher frequency of copulation than low-ranking dogs (who showed lower levels of affiliation to oestrus females). Conversely, male aggressiveness towards females during oestrous did not seem to function effectively to constrain female mate preference. Although, unlike other studies [80], [81], [86], we did not observe male dogs forcing females into mating, some males used aggression to try to intimidate females that refused to mate with them. Moreover, sometimes males showed aggressive behaviour towards females who were attempting to mate with other males. Females typically refused to mate with these aggressive males by actively avoiding, attacking or chasing them. In particular, our results show that old high-ranking females were more aggressive than young subordinate females in refusing male mount attempts.

Our results apparently disagree with other studies reporting that oestrous females disliked dominant males [80], [86]. However, in those studies the “dominance status” was assessed on the basis of physical power and aggressiveness, and a statistical evaluation of the transitivity of dominance relationships was not carried out. Instead, our results show that male aggressiveness towards females was inversely correlated to male social status, and it was mainly displayed by individuals that were frequently rejected by females, as reported previously [80], [86]. So, why should females reject low-ranking males, thus eliciting an aggressive response by them, and mate with high-ranking affiliative partners? One simple possibility is that oestrous females prefer to associate with high-ranking affiliative partners in order to reduce their vulnerability to harassment by the more aggressive low-ranking males (the “hired gun” hypothesis [106]). Aside from this consideration, it is plausible that dominance, as well as leadership and age which are positively correlated to dominance [65], [68], are good predictors of mate quality in domestic dogs. In this species the reproductive investment of females is higher than that of males, therefore they should select males on the basis of their quality. This is because males of high quality may increase the fitness of offspring (‘good genes’ hypothesis, [107]), and may also provide short-term benefits through indirect parental care (e.g. territorial acquisition, maintenance and defence; sentinel and anti-predator behaviours; caring for the pregnant mate through guarding and feeding). However, the relative importance of paternal contribution to raising pups and the possible relevance of the less evident indirect care has been poorly investigated in free-ranging domestic dogs. In some studies males were observed providing direct paternal care by defending puppies in the absence of the mother (by preventing the approach of strangers through vocalizations or even physical attacks; [88]), sleeping in close proximity to the mother and her litter, and playing with the pups when they became mobile [62]. Unfortunately, in our population we had limited possibilities of observing direct paternal investment because we were able to precisely locate puppies dens in a limited number of cases, and our presence around dens was discouraged by mothers through barking, growling and attacking. So, although more extensive studies are needed, it is possible that high-ranking and experienced male dogs might provide females with better direct and/or indirect paternal care, and their old age may also confirm viability in the current environment.

Our results also suggest that female intra-sexual competition affected male mate preferences. Using male mating effort as a proxy for male mate choice, we found that male dogs did not distribute this effort evenly among oestrus females. In fact, male courting effort and male-male competition for receptive females (measured as rate of mount attempts and proportion of interrupted mounts) appeared to be stronger in the presence of higher-ranking females, suggesting that males prefer to mate with dominant females. It is possible that male preferences for dominant females are functional because high-ranking females, based on our study, seem to have higher reproductive success than low-ranking ones. Rank-related reproductive asymmetries in bitches may result from competition for food resources in which high-ranking females usually prevail over subordinates [65], [70]. Another speculation is that the differential reproductive success may be a consequence of infanticide by dominant females. Although we never observed infanticide in our population of dogs, it has been documented in several canid species (e.g. *Canis lupus* [108], *Canis latrans* [109], *Canis aureus* [110]), and also in captive dingoes [111] that descend from domestic dogs [112]. Avoidance of intra-sexual competition may explain why, during our study and in others [60], some pregnant females dispersed from the pack before giving birth. Although the number of dispersing females in our study was too low to allow a statistical comparison, it is worth to note that they obtained a moderate reproductive success, whereas among resident females only the highest in rank had a considerable reproductive success (see Table 1). However, we cannot rule out that, in our study, some kind of undetected paternal investment by high-ranking males might have increased the reproductive success of their preferred females. So, an alternative interpretation of our results is that high-ranking females had higher reproductive success than low-ranking ones because high-ranking males provided them with paternal investment in exchange for being preferred as mates.

Finally, it is worth to stress that both female and male mate preferences may also partially reflect a strategy of inbreeding avoidance (see [77] for an example of inbreeding avoidance in wolves). For instance, although the highest-ranking male performed most copulations, he did not mate with young females. Overall, high-ranking old males showed a preference for high-ranking old females and vice versa. Inbreeding avoidance may partially account for these results because there was a certain probability that high-ranking and old male dogs, and especially the highest-ranking male, were the fathers of young low-ranking females. Furthermore, inbreeding avoidance may also contribute to explain why some females accepted the courtship of and mated with non-pack males.

### Comparison with the mating system of wolves

The domestic dog is a very recently evolved member of the genus *Canis*. Monogamy – exclusive mating between pair-bonded individuals – is rare in mammals but it is typical for wild members of the genus *Canis* [113]. The ancestor of domestic dogs, the wolf (*Canis lupus*), usually live in family packs consisting of a mated pair, their juvenile offspring, and adult helper offspring from previous years, whereas unrelated animals rarely associate with the group [76], [114], [115]. Usually, only a single pair within the pack breed and consistently lead group activities [76], [78], [116]. As long as offspring remain in their natal group, sexual maturation and mating are typically delayed until they disperse from the pack to seek for their own mates [115]. Moreover, in case subordinate wolves delay dispersal and reach sexual maturity in their natal pack, they are usually prevented from mating through active intervention by dominant animals [75], [117], [118]. All group members cooperate in raising puppies born to the dominant breeding female by providing allofeeding and other care to them [76], [119].

The social organization, the mating system and the reproductive biology of domestic dogs differ in several respects from those of their wild ancestors. Free-ranging dogs can form packs composed by related individuals, although they probably contain a higher proportion of unrelated animals if compared to wolves [62], [70], and also a higher number of sexually mature individuals of both sexes [60], [61], [62], [65], [67], [68], [70], [80]. Although several of these mature individuals usually breed in dog packs, we have shown for the first time in this paper that their reproductive performance can increase with their dominance rank, age, and tendency to lead pack movements. We believe that previous studies on groups of domestic dogs (reviewed in: [72], [74]) failed in documenting any social regulation of reproductive activities possibly because they either lacked detailed quantitative analyses of social interactions, or because the small number of females in the studied groups prevented them from ascertaining the statistical significance of reproductive asymmetries. In our opinion, it is likely that the social regulation of reproduction will operate in small groups of dogs as well, since dominance hierarchies can be found also in such groups [70]. Notably, a positive relationship between variables such as reproductive activity, dominance, age and leadership has also been found in wolf packs (e.g. [76], [78]), and this similarity suggests that some common organizing mechanism may contribute to shape the social organization of both species. According to our view, the main differences between the two species reside in the degree of reproductive suppression exerted by dominant animals over subordinates, and in the degree of cooperative breeding, that are usually higher in wolves (see also [70]).

The presence of multiple breeding individuals in dog packs might be explained functionally as an adaptive consequence of the domestication process. As suggested by several authors [58], [120], [121], unrestricted dog populations have adapted to scavenge from human refuses that are abundant and do not follow marked seasonal fluctuations. This continuous availability of food may have favored the loss of seasonal reproductive behavior, and may have allowed dogs to reproduce in their natal pack during the first year of their life, once they reach full body weight [58]. Furthermore, the abundance of food resources experienced by free-ranging dogs may have led to a decrease in the level of within group competition for food and in the degree of reproductive suppression of subordinates [70]. Conversely, unlike dogs, wolves are seasonal breeders and they rarely reach sufficient body size to reproduce until their second breeding season at the age of about 22 months, when they usually disperse from their natal pack [58], [122], [123], [124], [125]. However, wolf packs with multiple breeders can be found where food resources are unusually abundant and some individuals delay dispersal [76], [77], [112], [126], [127], [128], [129], [130], [131]. On the other hand, in a pack of feral dogs studied in an area with harsh weather and limited food availability, only one female gave birth during a two years period, and pup rearing apparently was shared by several group members [132]. Although detailed data about the social relationships among the members of this group are lacking, this example suggests that, under some extreme ecological conditions, dogs can form packs whose structure may be even more similar to that of wolf packs, and highlights the considerable social flexibility of this species.

The adaptation of dogs to exploit human refuse as a food resource may also account for the apparent reduction in allofeeding of lactating mothers observed in this species relative to wolves [58], [70]. Lactating female wolves spend a considerable portion of their time with pups at the denning site, and they are provisioned with food by the other pack members who perform most of the hunting [119], [133], [134], [135], [136]. Unlike wolves, most free-ranging dogs do not need to hunt to feed, and they can often rely on food sources that are presumably more predictable in terms of location and time than wolves' prey [70]. This usually allows lactating bitches to place their dens in the vicinity of human refuse, and thus to join their pack during feeding, while reducing the time during which pups are left alone [70]. So, the ecological conditions found in a domestic environment may have driven the evolution of an increased independency of bitches in raising their pups relative to female wolves.

Once we know that domestic dogs display mate preferences and that these are affected by the social relationships within the pack, we may ask how the former evolved. Notably, mate choice cannot evolve through artificial selection simply because the latter implies that human beings are those deciding which animals are allowed to mate. So, it may be hypothesized that 1) either mate choice evolved in wolves and was maintained in dogs by natural selection, or 2) that dogs evolved a different pattern of mate preferences by natural selection during the domestication process. The latter may also be plausible since, even nowadays, human beings seem to control the reproduction of a very limited portion of the global population of domestic dogs [58]. Consequently, in order to improve our understanding of the evolution of dogs, it is useful to compare their pattern of mate choice with that of their closest wild relatives. Some studies on captive wolf packs have reported mutual mate preferences between dominant males and females [137], [138], [139], [140], suggesting similarities between wolves and dogs. However, in another captive study, based on a more extensive behavioural data set [50], it was found that, although mating involved primarily high-ranking individuals of both sexes, this appeared to be mainly a consequence of the male preferences for high-ranking females and of dominant males limiting the sexual activity of subordinates. Conversely, females spread their sexual interest over several males and they did not consistently prefer high-ranking males. However, differences between studies may also be a consequence of different methods adopted by researchers in order to assess mate preferences. So, further studies are clearly needed, to confirm whether dogs have actually retained the mate preferences of their ancestors.

### Conclusions

To our knowledge, the present study provides the first clear evidence that an age-graded dominance hierarchy in a pack of free-ranging dogs affects several aspects of reproductive activities such as mate preferences, male copulation rate and female reproductive outcome. Dogs of both sexes displayed mate preferences for high-ranking partners, reflected in the differential distribution of affiliative signals, and old high-ranking dogs of both sexes showed a higher copulatory/reproductive performance. Overall, our results suggest that the social organization of pack-living free-ranging dogs may resemble that of wolves to a higher extent than previously thought. Further investigations of mate choice in both species may shed light on how their natural evolution diverged since the initiation of domestication.
